# Comprehensive protein profiling of synovial fluid in osteoarthritis following protein equalization

**DOI:** 10.1016/j.joca.2015.03.019

**Published:** 2015-07

**Authors:** M.J. Peffers, B. McDermott, P.D. Clegg, C.M. Riggs

**Affiliations:** † Comparative Musculoskeletal Biology, Institute of Ageing and Chronic Disease, University of Liverpool, Leahurst, Chester High Road, Neston, Wirral, CH64 7TE, UK; ‡ Hong Kong Jockey Club, Equine Hospital, Sha Tin Racecourse, New Territories, Hong Kong

**Keywords:** Synovial fluid, Equalization, Osteoarthritis, S100-A10, Neopeptide

## Abstract

**Objective:**

The aim of the study was to characterise the protein complement of synovial fluid (SF) in health and osteoarthritis (OA) using liquid chromatography mass spectrometry (LC-MS/MS) following peptide-based depletion of high abundance proteins.

**Design:**

SF was used from nine normal and nine OA Thoroughbred horses. Samples were analysed with LC-MS/MS using a NanoAcquity™ LC coupled to an LTQ Orbitrap Velos. In order to enrich the lower-abundance protein fractions protein equalisation was first undertaken using ProteoMiner™. Progenesis-QI™ LC-MS software was used for label-free quantification. In addition immunohistochemistry, western blotting and mRNA expression analysis was undertaken on selected joint tissues.

**Results:**

The number of protein identifications was increased by 33% in the ProteoMiner™ treated SF compared to undepleted SF. A total of 764 proteins (462 with≥2 significant peptides) were identified in SF. A subset of 10 proteins were identified which were differentially expressed in OA SF. S100-A10, a calcium binding protein was upregulated in OA and validated with western blotting and immunohistochemistry. Several new OA specific peptide fragments (neopeptides) were identified.

**Conclusion:**

The protein equalisation method compressed the dynamic range of the synovial proteins identifying the most comprehensive SF proteome to date. A number of proteins were identified for the first time in SF which may be involved in the pathogenesis of OA. We identified a distinct set of proteins and neopeptides that may act as potential biomarkers to distinguish between normal and OA joints.

## Introduction

Osteoarthritis (OA) is a joint disease characterised by alterations in the chondrocytes and loss of cartilage extracellular matrix (ECM)^1^, leading to biomechanical failure of articular cartilage. The molecular mechanisms involved in OA are not fully understood, with few currently validated markers for disease diagnosis and progression. Delayed diagnosis precludes preventive or timely therapeutic interventions. These limitations could be surmounted by enhanced knowledge of articular tissue metabolism and homoeostasis.

Synovial fluid (SF) is located in joint cavities and comprises a serum filtrate with additional contributions from surrounding tissues. As it is in direct contact with the OA-affected tissues it has been implicated as a contributor to OA pathophysiology. It also represents a potential source of disease-specific proteins that could both aid in the understanding of the pathogenesis of joint disease and act as disease biomarkers.

To date a number of mass spectrometry (MS)-based proteomic studies have been performed on both normal^2^ and diseased^3–5^ SF, with a variety of MS techniques^4–6^. In common with serum, SF contains many of high-abundant proteins (HAPs) with concentrations exceeding other proteins by estimated ten-orders of magnitude. Removal of HAPs aids in the detection of the lower abundance proteins which may be important in both OA pathogenesis and biomarker discovery. Immunoaffinity depletion has been used to remove these HAPs^5,7,8^; however large proteins, such as albumin, frequently act as carriers for other proteins which may themselves be significant^9^. Another method of compressing the dynamic range of protein but also enabling the entire proteome to be maintained is peptide-based depletion^10^. The method employs combinatorial hexapeptide ligand libraries immobilized on a solid phase matrix and has been used in serum proteomic studies^11^. This is the first time this approach has been used in SF proteomics.

The horse provides an accepted model for the study of arthropathies^12,13^ as it suffers similar clinical joint diseases to those seen in man^14–16^.

In this study we designed an innovative workflow to study normal and OA SF proteomic signatures. We used a well-documented cohort of skeletally mature horses, peptide-based depletion dynamic compression, LC-MS/MS with a high mass resolution instrument and label-free quantification. Additionally we identified several novel disease-associated peptide fragments (neopeptides).

## Methods

All chemicals were supplied by Sigma unless otherwise stated.

### SF sampling and procurement

SF, cartilage, subchondral bone and synovial membrane from the metacarpophalangeal joints of nine normal mean ± standard deviation (5.1 ± 0.5 years) and nine OA (6.5 ± 0.4 years) castrated male Thoroughbred horses at post-mortem. Samples were collected under the regulations of the Hong Kong Jockey Club with owner consent and stored at −80°C. OA diagnosis was based on macroscopic^17^, histological (modified Mankin)^18^ and synovitis scoring^19^ undertaken on joint tissues.

### SF preparation and protein depletion with ProteoMiner™

SF (1 ml) was treated with 1 μg/mL of hyaluronidase^5^. The supernatant was centrifuged through a 0.22 μm cellulose acetate membrane (Costar Spin-X, Corning, Tokyo, Japan) tube filter (5000× *g*/15 min) to remove insoluble material. Protein concentrations of SF were determined by Bradford assay (Thermo Scientific, Rockford, IL, USA).

For peptide-based depletion, 5 mg SF was bound to its own spin column containing ProteoMiner™ Small Capacity beads (Bio-Rad Laboratories, Hemel Hempstead, UK) with on-bead trypsin digestion.

### 1-D SDS PAGE

Following ProteoMiner™ treatment of one sample the eluate along with the flow-through (containing depleted proteins) and a sample of native SF were analysed by one dimensional sodium dodecyl sulphate polyacrylamide gel electrophoresis (1D-SDS-PAGE)^20^ to assess quantitative/qualitative differences in protein profiles.

### In-solution tryptic digestion and MS

Following *in-situ* trypsin digestion on ProteoMiner™ beads samples were treated with 1% (*w*/*v*) RapiGest (Waters, Manchester, UK) for 10 min at 80°C in 25 mM ammonium bicarbonate. To determine the effect of protein equalization of SF, equivalent protein from one sample of SF (undepleted sample) was digested by in-solution tryptic digestion^21^. LC-MS/MS analysis was performed on 2 μl aliquots of tryptic peptides equivalent to 1:10 dilution of the total digest using a 2 h gradient on a NanoAcquity™ ultraperformance LC (Waters, Manchester, UK) on line to an LTQ-Orbitrap-Velos (Thermo-Fisher Scientific, Hemel Hempstead, UK) as previously described^22^.

### Database search and protein identification

Raw spectra were converted to mascot generated files (mgf) (Proteome Discoverer software (Thermo, Hemel Hempstead, UK)). The resulting mgf files were searched against the *Equus caballus* database; Ensembl database for horse (*Equus caballus*; EquCab2.56.pep, (ftp://ftp.ensembl.org/pub/current_fasta/equus_caballus/pep/)) using an in-house Mascot server (Version 2.3.01) (Matrix Science, London, UK). Search parameters used were: enzyme; trypsin, peptide mass tolerances, 10 ppm; fragment mass tolerance, 0.6 Da; 1+, 2+ and 3+ ions; missed cleavages, 1; instrument type, ESI-TRAP. Modifications included were: fixed carbamidomethyl cysteine and variable; oxidation of methionine, lysine, proline.

### Label-free quantification

The Thermo raw files of the acquired spectra from in-solution tryptic digests were analysed by the Progenesis™ LC-MS software (Version 4, Nonlinear Dynamics)^21^. The top three spectra for each feature was exported from Progenesis-QI™ and utilized for peptide identification against the *E. caballus* database. Search parameters used were: 10 ppm peptide mass tolerance and 0.6-Da fragment mass tolerance; one missed cleavage allowed; fixed modification, carbamidomethylation; variable modifications, methionine oxidation, proline oxidation, and lysine oxidation. To maximize the number of quantifiable proteins but simultaneously use an acceptable false discovery rate (FDR), the peptide matches above an identity threshold were adjusted to give an FDR of 1% before the protein identifications being re-imported into Progenesis-QI™. Mascot determined peptides with ion scores of 20 and above, and only proteins with at least one unique peptide ranked as the top candidate were considered and analysed with only unique peptides being included. Statistical analysis was performed using transformed normalized abundances for one-way analysis of variance (ANOVA). All peptides (with Mascot score >23 and *P* < 0.05) of an identified protein were included, and the protein *P*-value (one-way analysis of variance) was then performed on the sum of the normalized abundances for all runs. Adjusted ANOVA values of *P* < 0.05 and additionally regulation of >2-fold or <0.5-fold were regarded as significant.

### Neopeptide identification

For neopeptide determination, MS data from the in-solution tryptic digests of normal and OA SF were analysed^22^. Filters for inclusion of neopeptides were as previously described^22^. Patterns of fragmentation were determined for aggrecan, biglycan, decorin, fibromodulin, and cartilage oligomeric matrix protein (COMP).

### Protein network analysis

Gene ontology (GO) was undertaken on the complete list of proteins identified in SF using the Panther Classification System version 9.0 (www.pantherdb.org)^23^ and Database for Annotation, Visualization and Integrated Discovery (DAVID) version 6.7^24^.

### Western blotting validation of S100A10 abundance

All validation studies were undertaken with an independent cohort but from the same population and with similar age ranges and macroscopic, Mankin's and synovitis scores. In order to validate the increased S100A10 abundance in OA SF, 50 μg total protein from normal and OA donors was used in western blot analysis^25^ using rabbit polyclonal to S100A10 (1:100 dilution, ABIN1386030, Antibodies online, Aachen, Germany) and a horseradish peroxidase conjugated goat anti-rabbit secondary antibody at 1:2000 and Western Lightning™ and Western Lightning Plus Chemiluminescence reagents (Perkin Elmer, Beaconsfield, USA). Blots were imaged using VisionWorksLS image acquisition software package and band densities were analysed using ImageJ 1.42.

### Immunohistochemistry of S100A10

Immunohistochemistry of cartilage and synovial membrane samples for S100A10 was undertaken^26^ using the primary antibody anti-S100A10 at 1:100 in blocking solution. The secondary antibody was a polyclonal anti-rabbit (Z0196, Dako UK Ltd., Ely, UK) at 1:100. No staining was observed in control experiments carried out with omission of the primary antibody and substitution with non-immune rabbit IgG (Abcam, Cambridge, UK). Digital image analysis was undertaken using ImageJ using the ‘threshold-color’ method^27^.

### RNA extraction and gene expression analysis

Cartilage, subchondral bone and synovial membrane from the metacarpophalangeal joint of selected normal (*n* = 8) and OA (*n* = 3) donors was collected into RNAlater and RNA extracted^12^. M-MLV reverse transcriptase and random hexamer oligonucleotides (both Promega, Southampton, UK) were used to synthesize cDNA from 1 μg RNA in a 25 μl reaction. PCR was performed on 1 μl 10× diluted cDNA, employing a final concentration of 300 nM of each primer in 20 μl reaction volumes on an ABI 7700 Sequence Detector using PrimerDesign 2X PrecisionTM SYBR Green Mastermix (Primer Design, Southampton, UK) using exon-spanning primers (Primer Design, Southampton, UK). Relative expression levels were normalised to GAPDH and calculated using the 2ˆDCt method^28,29^. Primers for equine GAPDH have been previously described^28^; S100A10 forward; GGGCTCACCATCGCATG, reverse; GTGGGAGGAATTGCTCAATG and CD109 forward; AAGGAGACAAGCGGTGAGAA, reverse; CGACAGACGAGTGACAACAG. RT-PCR analysis data was log_10_ transformed to ensure normal distribution and analysed using Student's *t* test.

## Results

### Macroscopic and histological assessment

Macroscopic scoring of samples from normal donors was 0 and OA donors were 2.3 ± 0.1SD. Normal donors had a Mankin's score 0.88 ± 0.4SD and OA donors 13.5 ± 1.4SD. Synovial membrane score from normal donors had a synovitis score of 1.2 ± 0.2SD and OA 1.7 ± 0.2SD.

### Protein depletion of SF

**Fig. 1:**
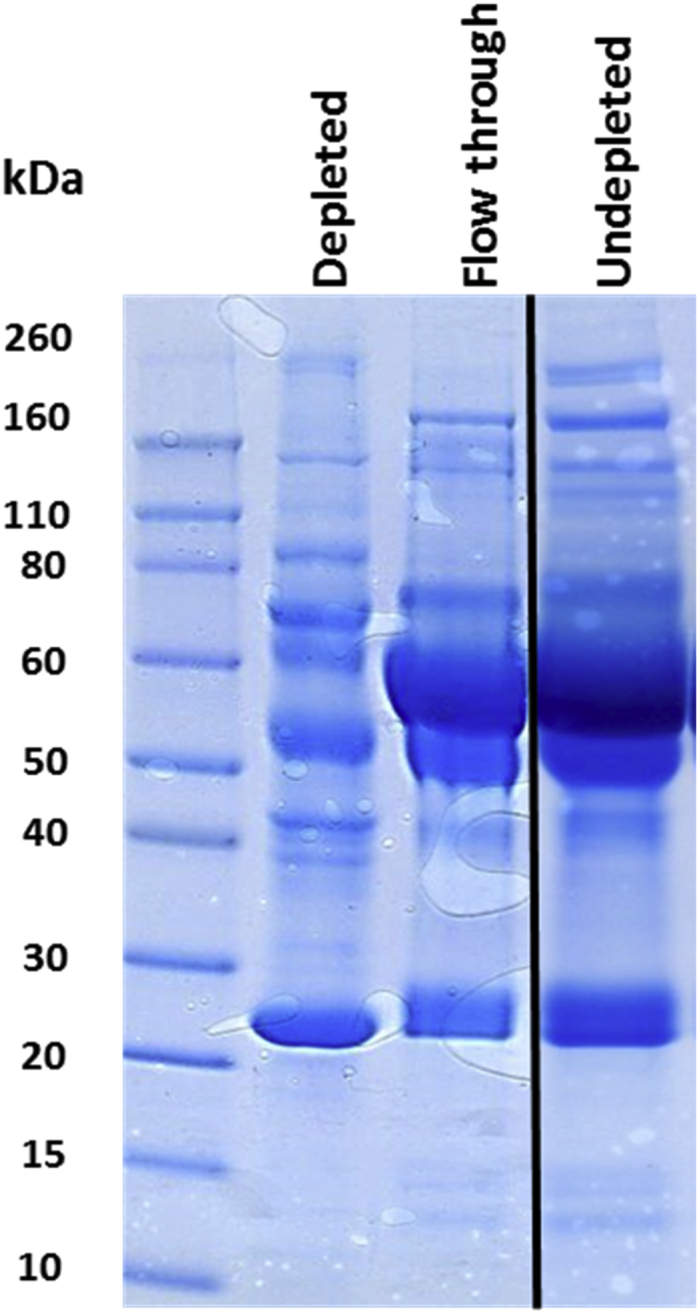
Coomassie Brilliant Blue stain of 1D SDS – PAGE gel of depleted and undepleted synovial fluid. All SF was pretreated with hyaluronidase as described in the methods. The lanes represent depleted (20 μg loading); 5 mg SF was loaded onto the ProteoMiner™ column and final eluted fraction represents depleted, flow through prior to elution; 60 μg load and undepleted; 60 μg native SF.

We used a peptide-based affinity method, to increase identification of low abundance proteins. To investigate the efficiency of this method we resolved non-depleted and depleted samples on a 1-D SDS PAGE gel to compare protein profiles. The peptide-based affinity method reduced the amount of albumin evident by a marked reduction in the 60 kDa albumin band (Fig. 1).

We then compared SF trypsin digests, with and without depletion, using LC-MS/MS and identified 204 proteins and 318 proteins (150 and 243 with significant Mascot score and ≥2 peptides) for native and ProteoMiner™ SF respectively; a 38% increase with ProteoMiner™. A list of proteins identified is in Additional File 1.

### Identification of proteins following ProteoMiner™ in normal and OA SF

A total of 764 proteins were identified in combined samples from SF; 462 with a significant Mascot score of >20 and ≥2 peptides. A global FDR of less than 3% was calculated by running a parallel search in a decoy database. Additional File 2 provides detailed information on the identification of each protein and corresponding Mascot scores.

For SF the total dataset with a significant Mascot score were either transformed to a non-redundant gene identifier list of the respective human homologues for DAVID analysis or input directly into Panther. Panther classified the proteins as cellular components (28%), cell organelles (26%), extracellular region (24%), ECM (15%) and macromolecular complex (7%).

**Fig. 2:**
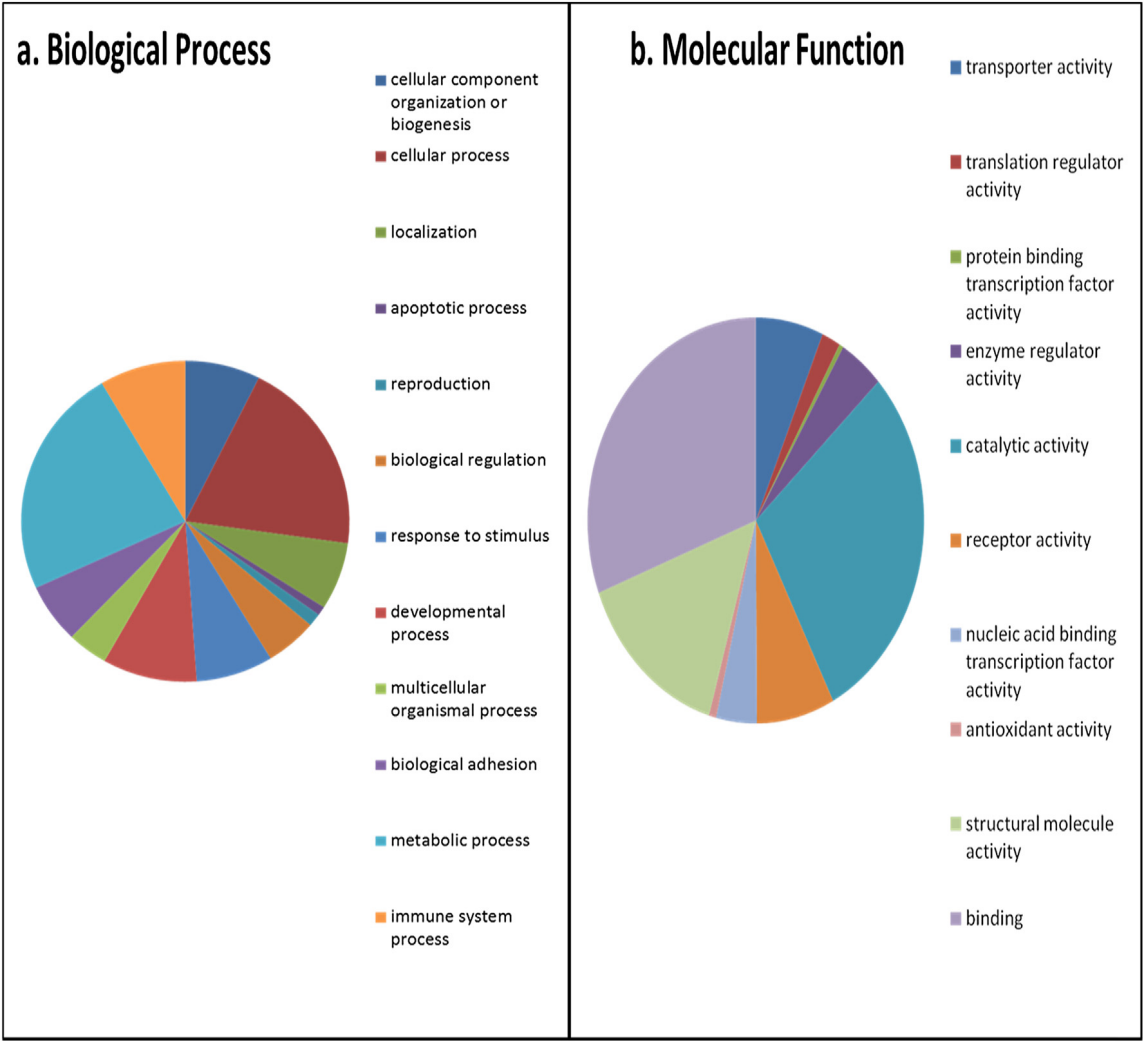
GO of SF using Panther. All proteins identified in SF were input into Panther protein classification software in order to determine the GO terms for (a) biological processes and (b) molecular function. Pie charts are representative of the percentage of proteins within each classification.

Figure 2 demonstrates the GO biological processes and molecular functions identified. Additional File 3 details protein class and cellular components identified. DAVID identified four significant Kegg pathways from the data set; complement and coagulation cascade, systemic lupus erythematosus, prion diseases and antigen processing and presentation (Bonferroni-adjusted *P*-values of 1.72E-23, 2.58E-14, 0.001, 0.04 respectively). Additional File 4 contains all results.

### Label-free relative quantification

In order to compare relative protein levels between normal and OA SF, samples were processed for LC-MS/MS and quantitative analysis undertaken with Progenesis-QI™. Principal component analysis of differentially expressed (DE) proteins with a greater than two-fold change in expression and *P* < 0.05 revealed that the proteins clustered according to OA status of the donor (principal component of 49%). Levels of 10 proteins significantly differed between normal and OA with ≥2 peptides. Six proteins were higher in OA SF and four proteins were lower in OA SF (Table I).

### Identification of ECM fragmentation patterns

A catalogue of OA related neopeptides in SF were identified for aggrecan, biglycan, COMP, decorin, fibromodulin, collagen alpha-1(I) and collagen alpha-3(VI) (Table II).

### Western blot validation of S100A10

**Fig. 3:**
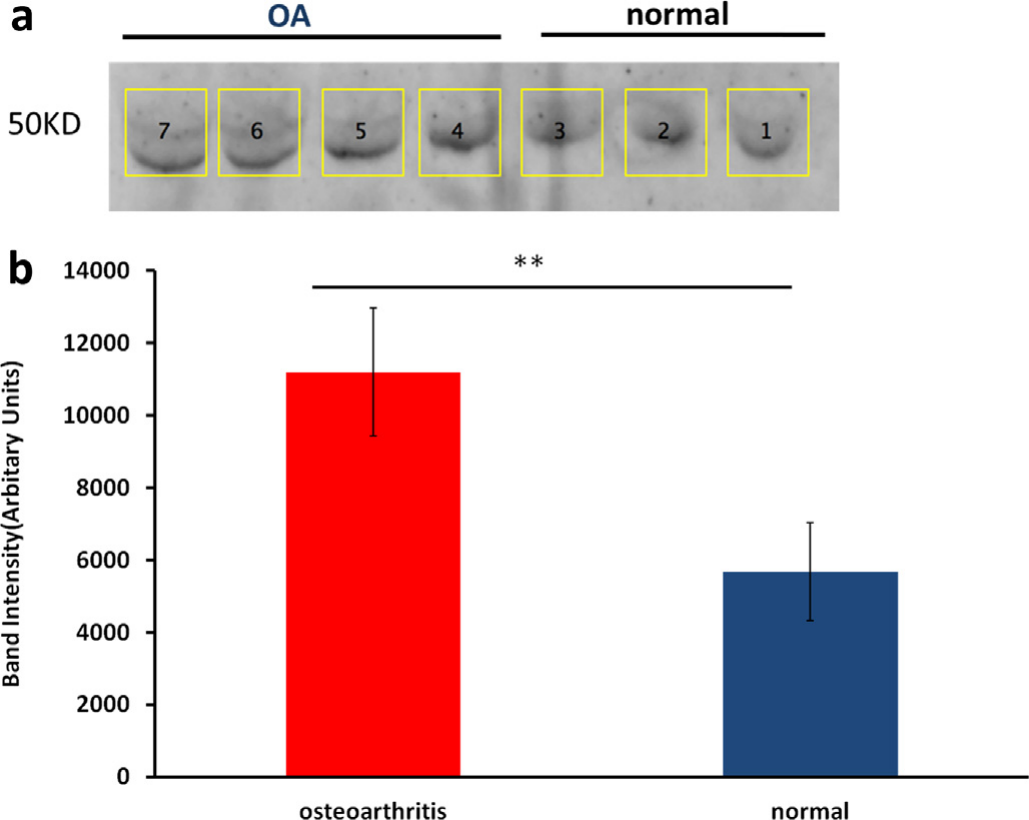
Increased abundance of S100A10 in OA SF. Representative image of western blot analyses of S100A10 (molecular weight 50 kD) in SF from five OA and three normal donors demonstrates an increase in S100A10 in OA. The blots are representative of eight OA and nine normal samples. Histogram represents mean pixel intensity of OA and normal S100A10 protein bands mean 95%CI, **P = 0.004 (Student's *t* test). Units are arbitrary.

We attempted to verify the results for the two proteins increased in SF with significant q-values; S100A10 and CD109. Unfortunately although antibodies were sourced with predicted reactivity against equine CD109, they did not work. We validated results for S100A10 using immunoblotting which confirmed that S100A10 appeared more abundant in OA SF (Fig. 3).

### Characterization of S100A10 in normal and OA cartilage and synovium

**Fig. 4:**
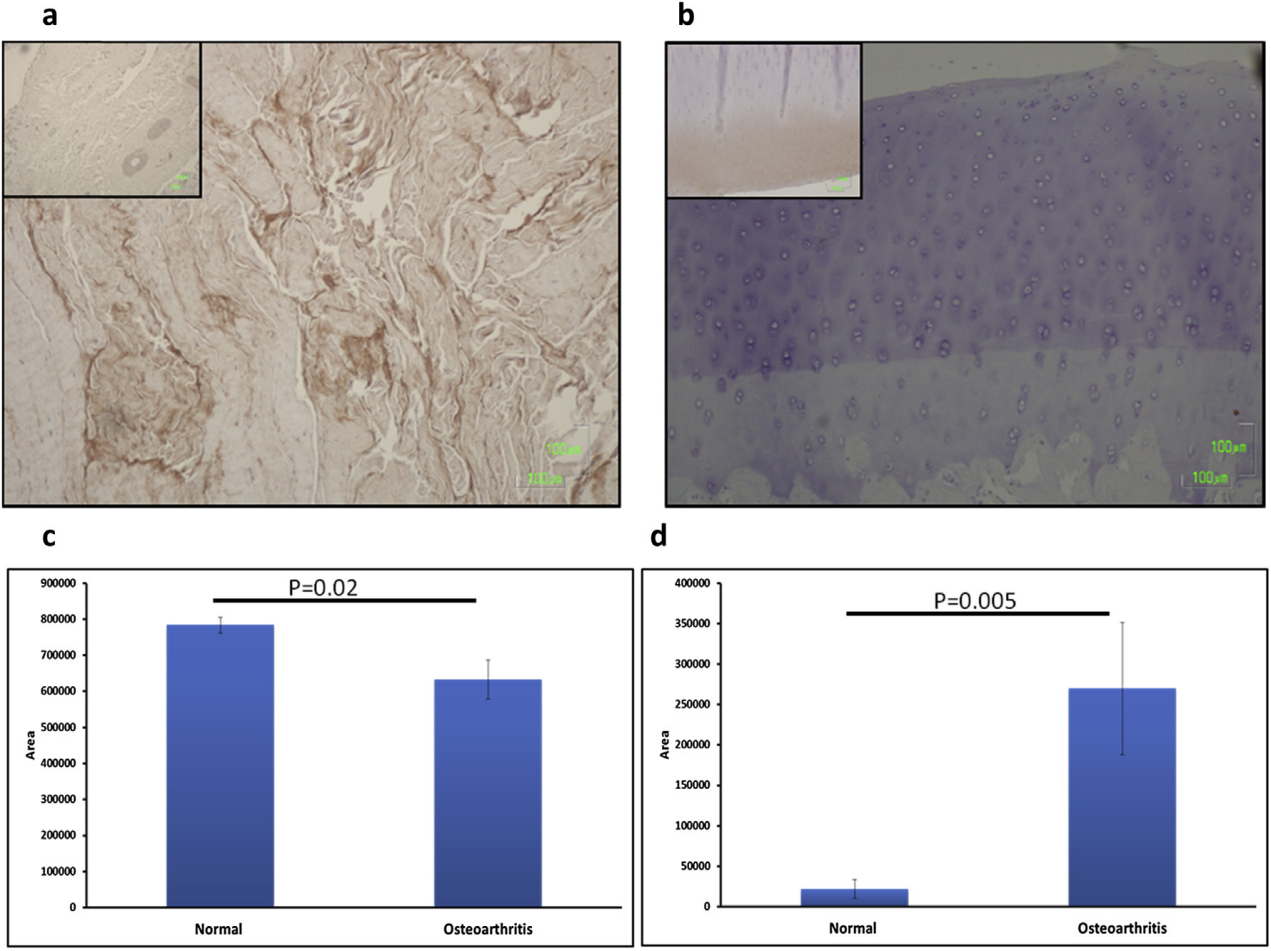
Localization of S100A10 in joint tissues from normal and OA horses. Cartilage with underlying subchondral bone and synovium obtained from normal and OA horses were probed with anti-S100A10 antibodies. Quantitative image analysis was undertaken using the ‘Threshold’ method in ImageJ. This ensures that only areas stained with a designated colour are selected for measurement; hence the *y*-axis in histograms (c) and (d) represents ‘area’. All images were captured at 20× magnification. (a) Synovial membrane sample of representative OA donor (inset) compared with a sample of a normal donor. (b) Cartilage with underlying subchondral bone from a representative OA donor (inset) compared with a normal donor. Histogram represents mean area for (c) synovial membrane for normal (*n* = 3) and OA (*n* = 2) and (d) cartilage with underlying bone for normal (*n* = 4) and OA (*n* = 3) mean 95%CI together with statistical significance of results (Student's *t* test).

Immunohistochemistry analysis of cartilage and synovium from normal and OA horses, using anti-S100A10, was undertaken to determine the strength of expression and its tissue distribution. Immunohistochemistry revealed reduced expression of S100A10 in a homogenous and diffuse pattern in OA synovium compared to normal synovium (*P* = 0.02; Student's *t* test) [Fig. 4(a) and (c)]. In contrast there was an increase in expression of S100A10 in OA compared to normal cartilage (*P* = 0.005; Student's *t* test) [Fig. 4(b) and (c)] demonstrated by increased diffuse staining in the tangential and transitional cartilage layers.

### S100A10 and CD109 gene expression in joint tissues

**Fig. 5:**
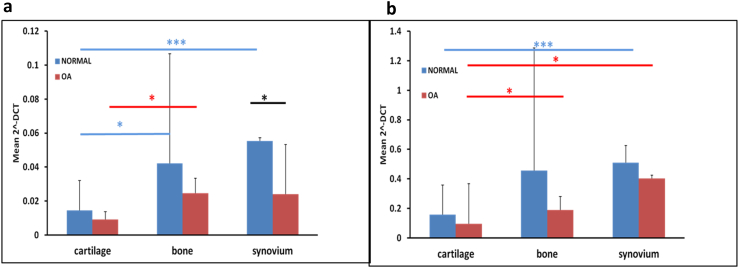
Gene expression at tissue and disease level of CD109 and S100A10. Cartilage, subchondral bone and synovium mRNA levels of (a) CD109, and (b) S100A10 in normal and OA donors. Data are represented as relative gene expression compared to GAPDH. Histograms represent means 95%CI. *P < 0.05, ***P < 0.001. Data were evaluated using Student's *t* test (normal *n* = 8, OA *n* = 3).

To determine whether the difference in S100A10 and CD109 we observed in OA SF was due to local production or represented extra-articular production we undertook gene expression analysis of cartilage, subchondral bone and synovium from normal and OA donors. There were significant tissue and disease differences in the relative gene expression of both CD109 and S100A10 (Fig. 5).

## Discussion

We have performed a comprehensive proteomic analysis of normal and OA SF identifying disease-related alterations to the proteins in OA. LC-MS/MS with high mass resolution instruments has enabled rapid advances in protein profiling of complex body fluids including SF^2,4^ which enables novel opportunities for gaining disease insight. Using label-free quantification, comparing age-matched controls, we identified a number of DE proteins in OA SF.

In order to minimise the high inter-donor variability previously reported^30^ we used post-mortem samples from a cohort of racehorses which were housed and trained at a single location, were age-matched (young, skeletally mature), of the same sex and we had access to macroscopically and histological details of the cartilage, subchondral bone, synovium. In addition we had full knowledge of their training and medical histories. This enabled us to choose control donors that had no pathological changes to the cartilage and limited synovium changes. In addition age-matching removed any age-related changes.

We developed a peptide-based affinity method (ProteoMiner™) which has been demonstrated to have a high level of reproducibility and consistency in terms of the species and quantities of proteins captured^31^. This method condenses the dynamic range of protein concentrations, whilst retaining a large proportion of the entire repertoire of proteins present in the initial sample, increasing resolving power of proteome characterisation. Moreover, as we were analysing equine SF there was a potential for poor-performance using affinity depletion methods based on human antigens. Previous work had demonstrated their usefulness in bovine serum studies^32^. It has been demonstrated that reliable differential quantitative data can be obtained from samples submitted to protein equalization^11,33^. However it is important to validate any findings with other methodologies. Whilst many studies have undertaken selected reaction monitoring (SRM) assays to validate results the peptide-based affinity method is now validated in many papers^11^. In one study 35 differentially expressed proteins were identified and the top 17 validated with SRMs and all agreed with the ProteoMiner™ data^34^. Furthermore a study has demonstrated that the enrichment (of medium and low abundance proteins) maintains quantitative information. The linearity of response to the addition of increasing amounts of serum amyloid A was demonstrated using MS^33^. Therefore we used other methods to validate and investigate our findings further. To estimate the efficiency of this method in SF proteomics we resolved non-depleted and depleted samples by 1D-SDS-PAGE. This confirmed depletion resulted in a more complex visual protein profile together with a reduction in some abundant bands. Furthermore we analysed non-depleted and depleted SF from the same donor using LC-MS/MS. This demonstrated a significant increase (38%) in the number of proteins identified following depletion, agreeing with serum comparative quantitative proteomics studies^35^. We identified 16 of the 20 proteins identified in a previous immunodepletion study^5^ in both depleted and undepleted samples. All except two of these had a reduced protein identification Mascot score and protein hit number in the depleted compared to undepleted sample. The change in Mascot generated protein hit number varied. For example albumin was the number one hit in undepleted SF and following treatment with ProteoMiner™ it became the sixth highest hit, whereas transferrin went from the second highest hit to the one hundred and thirty sixth highest hit.

In this study, following ProteoMiner™ treatment, LC-MS/MS with a high mass resolution mass spectrometer and the software Progenesis-QI™ we report one of the largest number of proteins identified in SF to date. This was despite the presence of HAP (five out of 10 of the top protein hits; complement C3, C4, albumin, apolipoprotein AI and B) in the results, similar to recent findings in normal porcine SF^2^. This was in part due to Progenesis-QI™ quantification algorithms that measures all detected peaks and then identifies them, which favours identification of low abundance peptides^36^. A recent publication found 575 proteins using an Isobaric Tags for Relative and Absolute Quantification (iTRAQ) study^37^ but this was a comparative study between OA and rheumatoid arthritis (RA) SF. Another study employing multiple fractionation strategies following immunodepletion identified 677 proteins in OA SF^8^.

The diversity of proteins identified in SF included collagenous and non-collagenous ECM proteins, proteases, signalling and immune response proteins. Our data was in agreement with other recent SF proteomics studies^3,5,8,37^ confirming the validity of our experimental workflow. Functional distribution of proteins by Panther highlights the breadth of proteins, dominated by intracellular and cell membrane proteins along with plasma proteins in SF. Functional annotation clustering using DAVID identified a number of Kegg pathways enriched. These included complement and coagulation cascade, which can be accounted for by the presence of a number of complement proteins, whilst systemic lupus erythematosus is represented by a number of complement proteins in addition to proteases such as cathepsin and cell surface antigens in SF.

We identified a number of DE proteins in OA. Of these the calcium-binding protein S100A10 (increased in OA SF) plus integral membrane protein 2B and cyclin D binding myb-like transcription factor 1 isoform 1 (reduced in OA SF) were identified for the first time. In our label-free quantification we were stringent in the filters used for input analysis; only proteins with an FDR of 1% and unique peptides were used for quantification. Additionally, we only targeted proteins for further study if differences had an FDR<0.05. This was undertaken in order to give the most robust results possible. Other SF studies have used 2D-DIGE^3^ or spectral counting^5,7,8^ to quantify, and the different proteomic workflows will account for differences evident between different studies.

Two proteins; CD109 and S100A10 were increased in OA SF with significant FDRs. CD109 is a transforming growth factor-β (TGF-β) co-receptor, released from the chondrocyte cell surface which inhibits TGF-β signalling by promoting TGF-β receptor internalisation and degradation^38^. CD109 has been detected in the peripheral circulation and SF as a component of CD146-positive lymphocytes in patients with numerous musculoskeletal diseases^39^. Previously it has also been identified in conditioned media of human articular chondrocytes^40^, bovine cartilage explants treated with interleukin 1β (IL1-β)^41^, and human OA cell lysates^42^. We attempted to undertake western blot validation and IHC in order to verify our MS results and localise CD109 but were unable to find a compatible antibody. Gene expression analysis identified that CD109 was expressed at the greatest level in synovium, followed by subchondral bone and cartilage. Furthermore there was a reduction in gene expression in OA synovium. Normal cartilage function is dependent on a narrow scale of bioactive TGF-β, with aberrations outside this range resulting in alterations in TGF-β signalling, resulting in abnormal cartilage function. As CD109 is an important factor in regulating TGF-β signalling, alterations in CD109 within the SF and synovium may play a role in OA pathogenesis. It could be that matrix degradation products or CD109 protein itself released from damaged cartilage in OA and the former of which lead to synovial inflammation may result in reduced CD109 expression in an attempt to promote TGF-β signalling. Further work is required to confirm this hypothesis.

We identified S100A10 in SF for the first time, although increased expression of S100A10 protein has been identified in OA and RA synovial fibroblasts^43^. The calcium binding protein S100A10, a member of the S100 family retains both intracellular and extracellular functions; including cytokine-like activities^44^. Several members of the S100 family have been identified in human articular cartilage, and their expression is upregulated in both OA and RA (reviewed by Yammani^45^). Other members of the S100 family have been demonstrated in OA^6,8^ and RA SF^6^. S100s have been indicated as potential biomarkers of other arthopathies^46^. Interestingly it has been established that some S100 members elicit a catabolic signalling pathway *via* receptors for advanced glycation end products leading to increased production of matrix-degrading enzymes; matrix metalloproteinases (MMPs) and a disintegrin and metalloproteinase with thrombospondin motifs (ADAMTSs), resulting in cartilage degradation and the development of arthritis^45^. S100A10 has been isolated in a complex with Annexin II (p36) which enables membrane localization of S100A10 by the formation of a heterotetramer (AIIt), interacting with plasminogen activator and plasminogen, stimulating its activation^47^. Through MAPK and NFκβ, AIIt induces IL1β, IL6 and tumour necrosis factor (TNF)α and consequently, silencing of S100A10 was demonstrated to reduce the secretion of TNFα, IL1β and Il10 in lipopolysaccharide stimulated chondrocytes^48^. Thus S100A10 has been proposed as a target for OA therapeutics. In our study we identified an increase in S100A10 protein in cartilage, but a reduction in the synovium in OA using IHC. It is therefore probable that the increase in S100A10 in SF evident in OA is due to an increase in its presence in cartilage, due to cartilage pathology in OA. This is then ‘shed’ into the SF on cartilage breakdown.

We also investigated OA-specific cleavage patterns in the ECM by assessing fragmentation patterns of specific matrix molecules in OA SF. A catalogue of ECM protein fragments, produced by fragmentation of the original peptide by enzymatic cleavage between two amino acids, which we have termed ‘neopeptides’, were identified in this study, and are likely due to specific proteinase cleavage. In our previous studies in equine ageing and diseased tendon we identified both tendinopathy and age-specific neopeptides^22^. In another study we identified age and OA related neopeptides in equine cartilage^13^. In this study all donor ages were similar and so we consider the neopeptides not to be age-specific. A number of previously documented, as well as novel neopeptides were identified here. These may represent proteolytic cleavage occurring as a consequence of pathological degradation, or they may be produced during sample processing as cell death can release intracellular proteases. The latter is unlikely as SF was collected rapidly post-mortem and immediately frozen. The neopeptides documented in this paper represent only those identified in young OA SF and it would be interesting to undertake further work on normal SF at different from donors of different ages in order to assess neopeptides produced by normal cartilage ageing as opposed to disease. A number of collagen-I and -VI neopeptides were identified. It is possible that the collagen-I neopeptides are from subchondral bone pathology which was evident in some samples on histological examination. It has been demonstrated that collagen VI gene expression is increased in OA and that it loses its pericellular location^49^.

## Conclusion

We demonstrated our novel workflow technique involving peptide-based equalisation followed by liquid chromatography coupled to a high mass resolution mass-spectrometer was appropriate for SF proteomics for both discovery and quantification. We discovered a distinct set of proteins that may act as potential biomarkers to distinguish between normal and OA joints. Further investigation postulated a role for S100A10 and CD109 in OA pathogenesis. Finally we also identified a catalogue of neopeptides in SF for the first time which may increase our understanding of OA pathogenesis and act as potential OA biomarkers.

## Figures and Tables

**Table I:** A number of differentially abundant proteins were identified by Progenesis™ LC-MS software between normal (A) and OA (B) SF using unique peptides only. All proteins with a >2-fold change and *P* < 0.05 in normalised abundance are shown

Highest mean condition	Accession	Protein	Role	Peptide count	Fold change	ANOVA
OA	NP_001157339.1	S100-A10∗	Calcium binding protein	2	2.2	0.000283
	XP_001498093.2	CD109 antigen∗	TGF β co-receptor	9	2	0.000391
	XP_001503419.1	Phospholipid transfer protein isoform 1	Lipid transfer protein	17	2.7	0.000981
	XP_001916113.1	Complement component C8 gamma chain	Constituent of the membrane attack complex	6	3.1	0.011525
	XP_001499636.3	Collagen alpha-1(I) chain	Fibrillar collagen	22	2.8	0.045984
	XP_001490837.3	Calsyntenin-1	Calcium binding protein	6	2.1	0.048534
Normal	XP_001489891.1	Integral membrane protein 2B	Processes amyloid A4 precursor protein	2	2.1	0.000676
	XP_001490429.3	Mannan-binding lectin serine protease 2	Protease	3	2.2	0.006101
	XP_001504425.1	Keratin, type II cytoskeletal 7	Blocks interferon-dependent interphase	8	2.2	0.011439
	XP_001488573.1	Cyclin D binding myb-like transcription factor 1 isoform 1	Transcriptional activator	2	6.2	0.024012

∗ In addition these proteins had a significant false-discovery rate adjusted *P* < 0.05.

**Table II:**
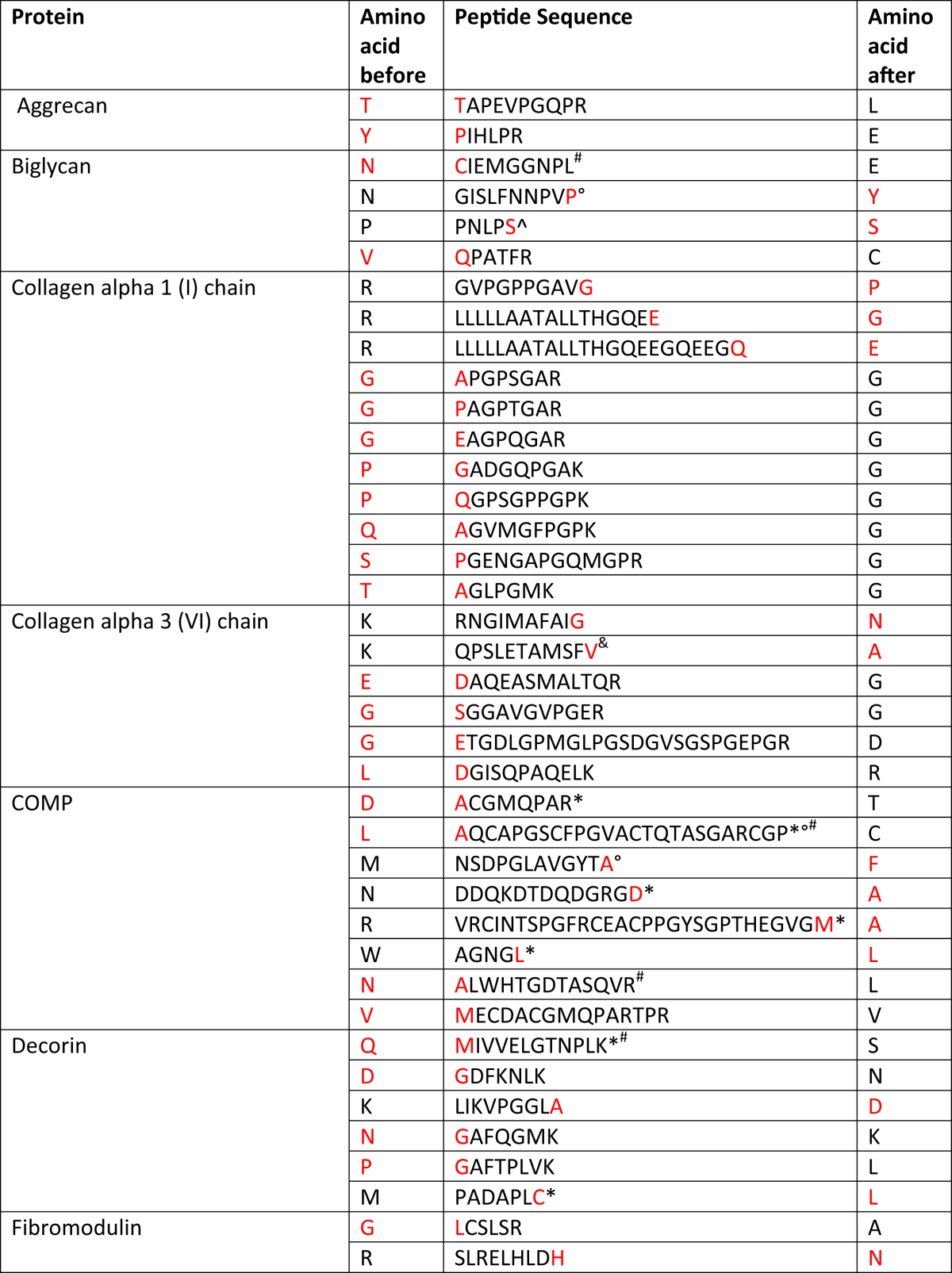
Neopeptides identified in OA SF. Neopeptides shown were identified by Mascot with a significant score in ≥2 SF but no normal donors together with a minimum ion score of 20 Amino acids shown in red correspond to the end of the neopeptide corresponding to a potential cleavage site. Amino acids before and after represent those preceding the peptide sequence and following the peptide sequence in the protein

* denotes found in previous interleukin-1 cartilage explant study. † denotes found in previous a disintegrin with thrombospondin motifs (ADAMTS4) study^50^. ‡ denotes found in previous matrix metalloproteinase 3 (MMP3) study^50^. § denotes previously identified in matrix-assisted laser desorption ionisation study^13^. ‖ denotes previously identified in equine tendon study^22^.

## Abbreviations

1D-SDS-PAGE: one dimensional sodium dodecyl sulphate polyacrylamide gel electrophoresis
2D-DIGE: two-dimensional electrophoresis
ADAMTS: a disintegrin and metalloproteinase with thrombospondin motifs
ANOVA: analysis of variance
COMP: cartilage oligomeric matrix protein
DE: differential expression
DAVID: The Database for Annotation, Visualization and Integrated Discovery
ECM: extracellular matrix
FDR: false discovery rate
GAPDH: glutaraldehyde dehydrogenase
GO: gene ontology
HAP: high abundance proteins
IL: interleukin
LC-MS/MS: liquid chromatography tandem mass spectrometry
MGF: mascot generated file
MMP: matrix metalloproteinases
OA: osteoarthritis
PPM: parts per million
RA: rheumatoid arthritis
RT-PCR: real-time polymerase chain reaction
SF: synovial fluid
SRM: selected reaction monitoring
TGF: transforming growth factor
TNF: tumour necrosis factor

## Supplementary data

Additional File 1. Proteins identified with and without ProteoMiner™ treatment. Description of data; Complete list of proteins identified in undepleted SF and following ProteoMiner™ treatment (depleted).

Additional File 2. The peptides and proteins identified in SF. Description of data; entire set of peptides and proteins with detailed information on the identification of each and corresponding Mascot scores.

Additional File 3. Panther results for entire SF dataset. Description of data; The entire data set of SF proteins were input into Panther. Results for protein class, molecular function, biological process and cellular components are tabulated.

Additional File 4. DAVID results for entire SF dataset. Description of data; Results following input of entire Sf dataset into DAVID. Table shows all results for functional annotation clustering with significant Kegg pathways highlighted (Bonferroni corrected *P* value <0.05).
